# Rapid Determination of Antibiotic Resistance in *Klebsiella pneumoniae* by a Novel Antibiotic Susceptibility Testing Method Using SYBR Green I and Propidium Iodide Double Staining

**DOI:** 10.3389/fmicb.2021.650458

**Published:** 2021-06-09

**Authors:** Yabin Zhang, Weihua Fan, Chunhong Shao, Jiajia Wang, Yan Jin, Jing Shao, Ying Zhang, Yong Wang

**Affiliations:** ^1^Department of Laboratory Medicine, Shandong Provincial Hospital Affiliated to Shandong First Medical University and Shandong Academy of Medical Sciences, Jinan, China; ^2^Department of Laboratory Medicine, Shandong Provincial Hospital Affiliated to Shandong University, Jinan, China; ^3^State Key Laboratory for the Diagnosis and Treatment of Infectious Diseases, Collaborative Innovation Center for Diagnosis and Treatment of Infectious Diseases, The First Affiliated Hospital, Zhejiang University School of Medicine, Hangzhou, China

**Keywords:** *Klebsiella pneumoniae*, antibiotic resistance, antibiotic susceptibility testing, SYBR Green I, propidium iodide

## Abstract

Due to the broad-spectrum antibiotic usage and empirical treatments, the pathogenic bacterium, *Klebsiella pneumoniae*, has shown extremely high detection rates at hospitals with an increasing antibiotic resistance. Therefore, rapid detection of the antibiotic resistance is urgently required and essential for effective treatments. In this study, we evaluated the performance of a newly developed method for ultra-rapid detection of antibiotic resistance in 30–60 min in *K. pneumoniae* by using the SYBR Green I and propidium iodide (PI) staining. A total of 100 clinical isolates were tested for antibiotic resistance using four different antibiotics (ceftriaxone, cefepime, meropenem, and ciprofloxacin). The results showed that the SYBR Green I/PI rapid antibiotic susceptibility test (AST) could reliably detect antibiotic resistance to the four drugs in 60 min, and the results were highly concordant with the conventional AST (i.e., Kirby-Bauer method and broth microdilution method) for detection of ceftriaxone, cefepime, meropenem, and ciprofloxacin resistance with a high accuracy of 99, 96, 96, and 93%, respectively. Therefore, the rapid AST established in our study helps to enable targeted therapy to save lives and reduce the empirical use of antibiotics and ultimately the health and economic burdens of antibiotic resistance.

## Introduction

*Klebsiella pneumoniae* is a non-spore-forming Gram-negative enterobacterium. This bacterium has become the most frequently detected pathogenic organism other than *Escherichia coli* (Hu et al., 2019) causing severe pneumonia, sepsis, and other systemic infections often involving multiple organs. It is highly prevalent in both infants, elderly people, and patients with diabetics, cancers, and long-term antibiotic use (Podschun and Ullmann, 1998). In recent years, *K. pneumoniae* infections have shown an increasing resistance with long-term use of antibiotics, including particularly the third-generation cephalosporins and carbapenems (Hu et al., 2019; Kang et al., 2020). Studies have shown that the fatality rate of carbapenem-resistant *K. pneumoniae* is 20–40% higher than that of the sensitive *K. pneumoniae* (Nordmann et al., 2009; Iredell et al., 2016). Infections with carbapenem-resistant pathogens require prolonged hospitalization with increased treatment costs, boosting the mortality rate associated with infectious diseases and severe financial and psychological stresses for both patients and their families (Lee et al., 2017; Makhado et al., 2018; Rodríguez-Baño et al., 2018; Ting et al., 2018).

To date, the antibiotic resistance in *K. pneumoniae* has become a severe threat to human public health worldwide (Tamma et al., 2017). Conventional antibiotic susceptibility test (AST) methods for *K. pneumoniae* have several disadvantages in practice, i.e., relying largely on bacterial growth and often taking a long time (e.g., 16–24 h) to generate the results, which can inevitably contribute to the risk of transmission and development of antibiotic-resistant *K. pneumoniae*. Evidently, it is necessary to establish a rapid and sensitive assay to detect the pathogenic bacteria and their antibiotic resistance (Price et al., 2014).

Recently, we developed a highly efficient and rapid method to detect antibiotic resistance in *K. pneumoniae* within 60 min using SYBR Green I and propidium iodide (PI) double staining (Feng et al., 2018). The SYBR Green I stains the nucleic acids of living cells to produce green fluorescence, while the PI is a membrane-impermeant dye that stains the nucleic acids in dead or damaged cells to generate red fluorescence (Boulos et al., 1999; Feng et al., 2014a; Duedu and French, 2017). Therefore, this SYBR Green I/PI double staining method determines the antibiotic resistance by detecting the changes in green and red fluorescence to evaluate the antibiotic resistance or sensitivity, respectively. To date, the rapid SYBR Green I/PI AST has been evaluated in several pathogenic bacteria, including *E. coli*, *Staphylococcus aureus*, *Borrelia burgdorferi*, and *Mycobacterium tuberculosis* (Feng et al., 2014a,b, 2016, 2018) with stabilized results obtained for both fast- and slow-growing pathogens in 60 min (e.g., *E. coli*) and 16 h (e.g., *M. tuberculosis*), respectively. Although it is currently a testing assay investigated in the laboratory, the results have demonstrated that this method has shown significant potential in the extremely rapid detection of antibiotic resistance. Due to the highly variable nature of the genetic background of different clinical isolates and the strict criteria for clinical diagnosis, it is necessary to evaluate and optimize this method in clinical strains of bacteria.

In this study, we evaluated the SYBR Green I/PI AST method for ultra-rapid detection of antibiotic resistance to different antibiotics in clinical isolates of *K. pneumoniae*. The results showed a high degree of reliability and validity of the SYBR Green I/PI AST method for ultra-rapid detection of antibiotic resistance in *K. pneumoniae* clinical isolates with the current conventional ASTs in the Clinical and Laboratory Standards Institute (CLSI) guidelines. This study provides strong evidence to support the application of the ultra-rapid SYBR Green I/PI AST method in the clinical detection of antibiotic resistance, which will help guide the appropriate use of antibiotics for improved treatment and prevention of the spread of antibiotic-resistant bacteria.

## Materials and Methods

### Bacterial Strains, Culture Media, Chemicals, and Antibiotics

A total of 100 *K. pneumoniae* clinical isolates were collected at the Shandong Provincial Hospital, Jinan, China, from May 2019 to June 2020. The bacterium *K. pneumoniae* ATCC700603 was used as a control in this study. The cryopreserved clinical strains were inoculated on the Columbia blood agar medium and cultivated for 18–24 h at 37°C to activate the strains. Then, a fresh colony was picked from the blood agar medium, inoculated into the Luria-Bertani (LB) medium, and incubated at 37°C with shaking to reach an OD_600_ value of 0.2 measured using a turbidimeter (BioMérieux Densichek) or UV/Vis spectrophotometer (Yoke Instrument N5000). Cultures prepared in saline with different incubation times were counted for colonies on blood agar plates. Four types of antibiotics (ceftriaxone, cefepime, meropenem, and ciprofloxacin) purchased from the National Institute for Food and Drug Control (Beijing, China) were dissolved in appropriate solvents to obtain the stock solutions, filter-sterilized by 0.2 μm filter, and stored at −20°C before use.

### Conventional Antibiotic Susceptibility Assay

The minimum inhibitory concentrations (MIC) of ceftriaxone, cefepime, and ciprofloxacin for 100 clinical isolates of *K. pneumoniae* were determined by the broth microdilution method (Bio-Kont, China), while the resistance to meropenem was assessed by the Kirby-Bauer (K-B) method based on the measurement of its inhibition zone diameter in millimeter (mm). Briefly, the diluted suspension was inoculated into antibiotic-containing microplates or the drug sensitivity paper disks were pasted onto M-H plates coated with a 0.5 McFarland bacterial suspension which were incubated at 35°C for 16–24 h. Vitek 2 N335 was used for the retesting of the strains that gave inconsistent results of the conventional microdilution methods and the SYBR Green I/PI AST method (see below). All results obtained for antibiotics were interpreted in accordance with the 2019 guidelines of CLSI M100.

### SYBR Green I/PI Staining

Both SYBR Green I (10,000 × stock, Invitrogen; 30 μl) and PI (20 mm, Sigma; 10 μl) were diluted in 1,000 μl of distilled water and thoroughly vortexed to prepare a double staining dye for *K. pneumoniae*. In each reaction, 100 μl of culture with 10 μl of SYBR Green I/PI staining mixture was added and incubated for 20 min in the dark at room temperature. The fluorescence intensity was measured using a SpectraMax Gemini EM fluorescence microplate (Molecular Devices, CA, United States) with the excitation wavelength set at 485 nm and the green and red emission wavelengths at 538 nm and 612 nm, respectively.

The proportion of live bacteria was calculated based on the linear regression equation (i.e., the standard curve plotted between live bacteria and S values). To generate the standard curve, live and dead bacteria were mixed in seven different ratios (0%/100%, 20%/80%, 35%/65%, 50%/50%, 65%/35%, 80%/20%, and 100%/0%) of *K. pneumoniae* suspension in the 96-well plates. After adding the SYBR Green I/PI dye mixture, the green (G) and red (R) fluorescence intensity values were measured with SpectraMax Gemini EM fluorescence microplate for different proportions of live and dead bacteria. To reduce the effect of cell concentration on the fluorescence intensity, an S value was introduced to microplate fluorescence intensity with the following formula: $S\;=\;\left(G\;-\;\mathrm{3R}\right)/\left(G\;+\;\mathrm{3R}\right)$. The linear regression equation for live bacteria and S value was obtained by the least-square fit analysis. Specimens of *K. pneumoniae* were examined and imaged on a Leica laser scanning confocal microscope with the fluorescence intensity quantification performed by the Leica Application Suite X (Germany) and SoftMax Pro software.

### SYBR Green I/PI Staining for Rapid Antibiotic Susceptibility Testing of *Klebsiella pneumoniae*

Overnight stationary phase cultures of the isolated bacterial strains were diluted with a fresh medium for the OD_600_ value to reach 0.2. The dilution in fresh culture medium would allow the bacteria to become more active metabolically so they would respond to antibiotic treatment. To identify the optimal antibiotic exposure concentration, six resistant and six sensitive strains to ceftriaxone, cefepime, meropenem, and ciprofloxacin were treated with different concentrations of these four types of antibiotics, respectively. Subsequently, the culture mixture containing the antibiotics of the optimal concentrations was incubated for 40 min at 37°C and then stained with the SYBR Green I/PI staining solution for 20 min in the dark at room temperature. The proportion of viable bacteria was determined as described above. The same four types of antibiotics were applied to qualitatively determine the effect of antibiotics in rapid AST for *K. pneumoniae*. Cultures of clinical isolates were added to the 96-well plates with each well containing 10 μl pre-diluted antibiotics of incremental concentrations and 100 μl cultures of *K. pneumoniae* at its stationary phase as the treatment groups, while the control group was cultured with 10 μl of water. The final volume in each well was adjusted to 200 μl using LB medium. The microplates were sealed and incubated for 40 min in an incubator at 37°C; then 10 μl of the SYBR Green I/PI dye mixture was applied to both the treatment and the control groups. Then, the plates were incubated for 20 min in the dark at room temperature and fluorescence was measured using the fluorescence microplate reader as described above and the S values were calculated for both the treatment and the control groups. The percentage of viable bacteria (V) was determined using a standard curve as described above, and the relative percentage of surviving viable bacteria (RS) after drug exposure was calculated by the formula $\mathrm{RS}=V_{\mathrm{treated}}/V_{\mathrm{control}}$. All experiments were repeated three times.

### Construction of Receiver Operating Characteristic Curve

A receiver operating characteristic (ROC) curve based on all strains using RS values and their phenotypic sensitivity was constructed to validate the SYBR Green I/PI AST method in evaluating the bacterial viability. The optimal cutoff point and the diagnostic values of the SYBR Green I/PI staining for detecting the antibiotic resistance of *K. pneumoniae* clinical isolates were determined based on the ROC curve.

### Statistical Analyses

The student’s *t*-test was applied to evaluate the differences between the treatment and the control groups. Significant differences were determined at *p* < 0.05. Data were expressed as the mean ± standard deviation (SD). Statistical analyses were conducted using Excel and GraphPad Prism 8 software.

## Results

### Conventional Antibiotic Susceptibility Tests on *Klebsiella pneumoniae* Clinical Isolates

To evaluate the accuracy of the SYBR Green I/PI AST method, a total of 100 clinical isolates of *K. pneumoniae* were randomly selected for conventional ASTs (i.e., the K-B and the broth microdilution methods) with four types of commonly used antibiotics (ceftriaxone, cefepime, meropenem, and ciprofloxacin) in clinical settings. Strain resistance was determined by the breakpoint range (i.e., the MIC or the inhibition zone diameter). The result showed that a total of 43/57, 42/58, 41/59, and 47/53 isolates were resistant/sensitive to ceftriaxone, cefepime, meropenem, and ciprofloxacin, respectively (Table 1).

**Table 1:** Conventional susceptibility tests of a total of 100 *Klebsiella pneumoniae* clinical isolates and the diagnostic efficiency of the antibiotic resistance determined by the SYBR Green I/PI staining.

Antibiotics	Conventional antibiotic susceptibility tests (ASTs)						SYBR Green I/PI		
	Breakpoint for sensitivity or resistance		K-B		MIC		Sensitivity	Specificity	Accuracy
			R	S	R	S			
Ceftriaxone	≤1	≥4	–	–	43	57	98.25% (56/57)	100% (43/43)	99% (99/100)
Cefepime	≤2	≥16	–	–	42	58	93.10% (54/58)	100% (42/42)	96% (96/100)
Ciprofloxacin	≤0.25	≥1	–	–	47	53	88.68% (47/53)	97.87% (46/47)	93% (93/100)
Meropenem^*^	≥23 mm	≤19 mm	41	59	–	–	96.61% (57/59)	95.12% (39/41)	96% (96/100)

K-B, Kirby-Bauer method; MIC, minimum inhibitory concentration; R, resistant; and S, sensitive.

* Susceptibility or resistance to meropenem was assessed by the Kirby-Bauer (K-B) method based on measuring the diameter of inhibition zone in millimeter (mm). The diameter of inhibition zone >23 mm is defined as susceptible while that of <19 mm is resistant.

### Monitoring Growth of *Klebsiella pneumoniae* Using the SYBR Green I/PI Staining

**Figure 1:**
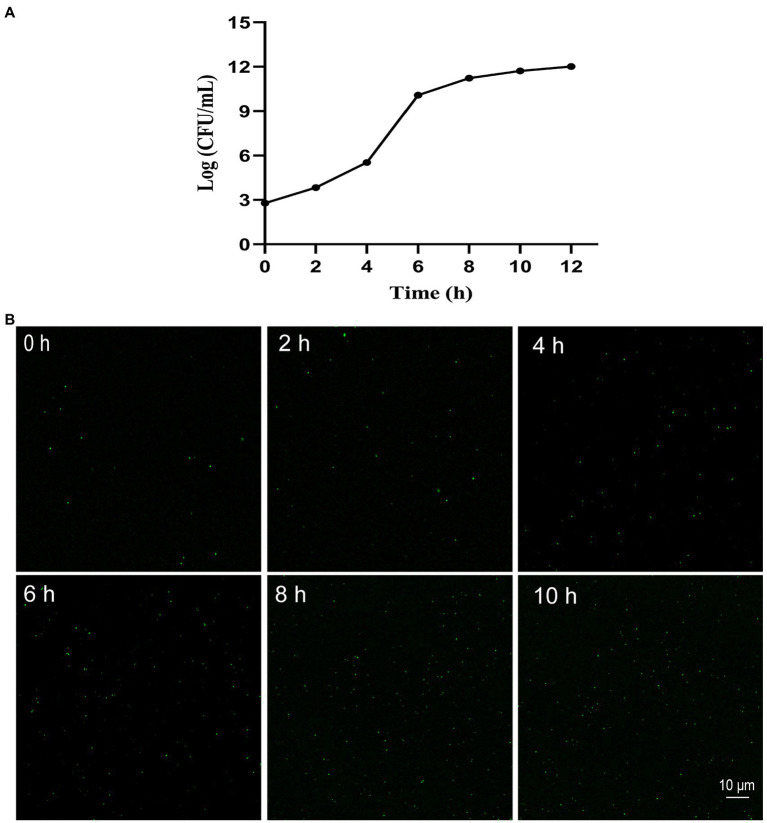
Growth of *Klebsiella pneumoniae in vitro* with growth curve **(A)** and the representative fluorescence images (400 × magnification) of SYBR Green I/PI staining at 0, 2, 4, 6, 8, and 10 h, respectively **(B)**.

Cultures of the quality control *K. pneumoniae* at different incubation times (0, 2, 4, 6, 8, 10, and 12 h) with an OD_600_ value of 0.2 treated with SYBR Green I/PI staining were visualized under a laser scanning confocal microscope. The results showed that the number of bacteria increased with the incubation time with the logarithmic and stable growth phases identified at 2–6 h and 8–12 h, respectively (Figure 1A). To verify the changes in the number of bacteria, the fluorescence images were taken at different incubation times (Figure 1B). The results measured on the fluorescence images were consistent with those derived by the above culture count.

### Evaluation of *Klebsiella pneumoniae* Viability Using the SYBR Green I/PI Staining

**Figure 2:**
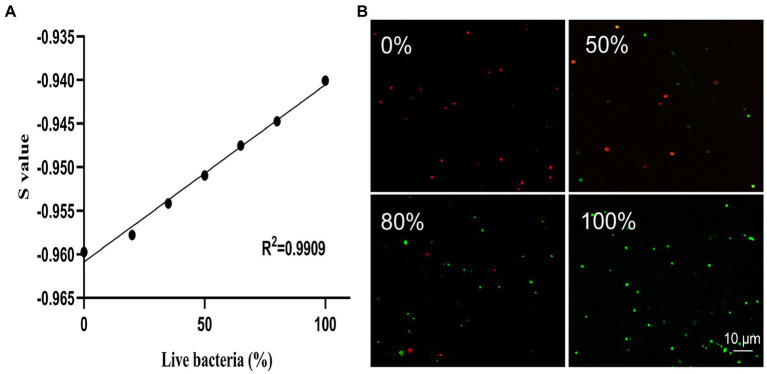
The linear regression relationship between the percentage of live bacteria and the S value based on the SYBR Green I/PI staining of *K. pneumoniae* measured using a fluorescence reader **(A)** and the representative images of fluorescence microscopy at different proportions (0, 50, 80, and 100%) of live bacteria **(B)**. The green and red dots represent the live and dead cells of *K. pneumoniae*, respectively.

The mixtures of live bacteria and dead cells with different ratios (0%/100%, 20%/80%, 35%/65%, 50%/50%, 65%/35%, 80%/20%, and 100%/0%) were stained with SYBR Green I/PI and the intensities of green and red fluorescences were measured using SpectraMax Gemini EM fluorescence reader to generate the standard curve plotted between the number of live bacteria and the S values (Figure 2A). The results showed that the S values and the percentages of live cells of *K. pneumoniae* exhibited a strong linear relationship represented by the regression equation of $V=\left(S+0.9609\right)/0.0002$ with an *R*^2^ value of 0.9909. The results of SYBR Green I/PI staining on the bacterial cultures with different proportions of live bacteria (0, 50, 80, and 100%) measured by the laser scanning confocal microscopy (Figure 2B) were consistent with those obtained by the fluorescence reader.

### Establishing the Antibiotic Concentrations to be Used for Rapid Susceptibility Testing of *Klebsiella pneumoniae* Using the SYBR Green I/PI Staining

**Figure 3:**
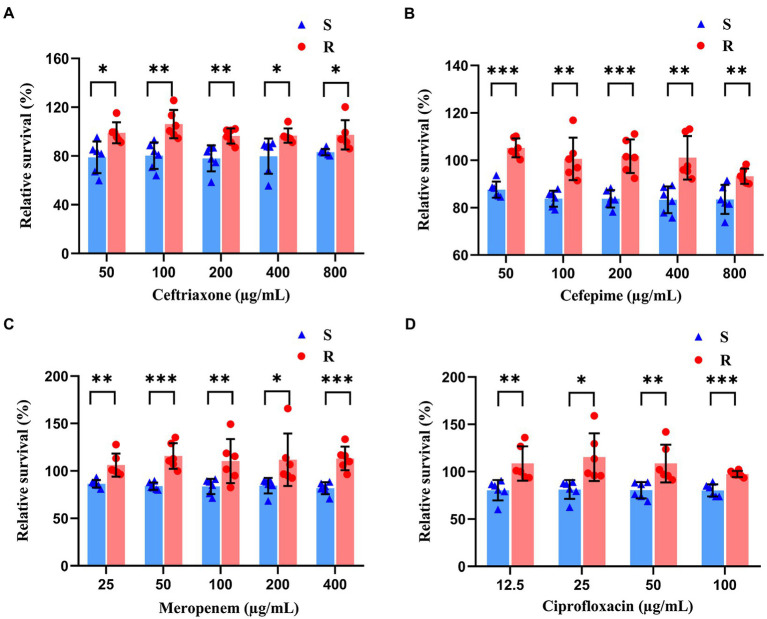
Antibiotic effects revealed by SYBR Green I/PI staining on both sensitive and resistant strains of *K. pneumoniae* treated with ceftriaxone **(A)**, cefepime **(B)**, meropenem **(C)**, and ciprofloxacin **(D)** of incremental concentrations for 40 min. The symbols of red dots and blue triangles represent the resistant and sensitive strains of bacteria, respectively. Statistical differences set at *p* < 0.05, *p* < 0.005, and *p* < 0.0005 are indicated by symbols *, **, and ***, respectively.

To optimize the conditions for antibiotic effects on *K. pneumoniae*, stationary phase *K. pneumoniae* (i.e., 10 h of culture) was selected for antibiotic exposure studies. Sensitive and resistant strains were distinguished based on the relative percentage of live cells after the drug exposure. Results showed that after the treatment of antibiotics with incremental concentrations for 40 min, the relative percentages of live cells were significantly lower in the sensitive groups than those of the resistant groups (Figure 3). Statistical analysis showed that the greatest significant differences between resistant and sensitive strains were found at concentrations of 100 μg/ml (ceftriaxone), 50 μg/ml (cefepime), 50 μg/ml (meropenem), and 100 μg/ml (ciprofloxacin). Therefore, the concentrations of 100, 50, 50, and 100 μg/ml were selected for ceftriaxone, cefepime, meropenem, and ciprofloxacin, respectively, in the following experiments.

**Figure 4:**
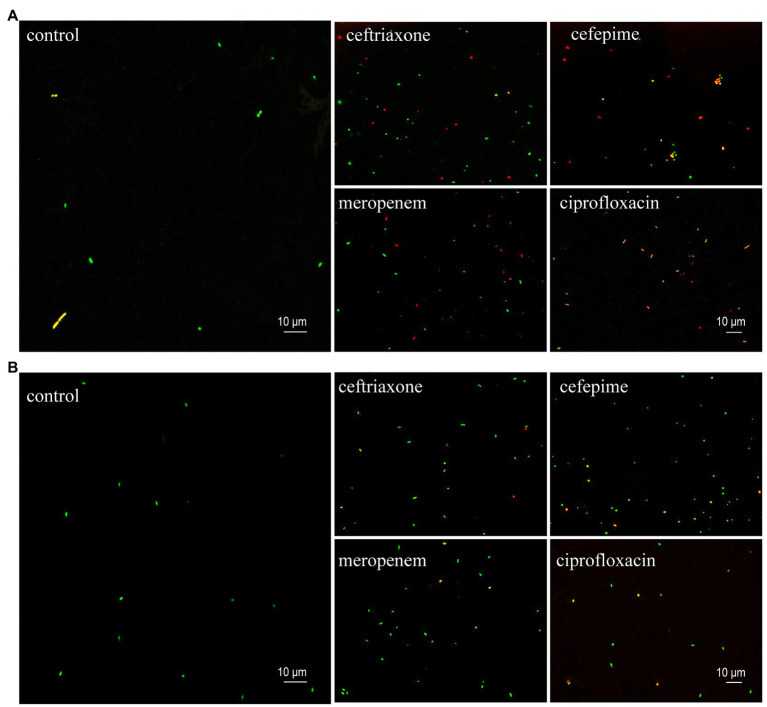
Representative images (630 × magnification) of SYBR Green I/PI staining of the sensitive **(A)** and resistant **(B)** clinical isolates of *K. pneumoniae* at their stationary phases treated with four types of antibiotics (i.e., ceftriaxone, cefepime, meropenem, and ciprofloxacin). The control groups contain no antibiotics. The green and red dots indicate the live and dead bacteria, respectively.

The results of AST of *K. pneumoniae* clinical isolates detected by the SYBR Green I/PI staining and observed using the confocal microscopy were consistent with those based on the fluorescence plate reader, indicating that the sensitive and resistant strains could be distinguished based on the antibiotic concentrations used in this study (Figure 4). Under the exposure of antibiotics, the sensitive strains showed the decreased intensity of green fluorescence with increased intensity of red fluorescence (Figure 4A), while the resistant strains showed the increased intensity of green fluorescence and decreased intensity of red fluorescence (Figure 4B).

### Antibiotic Susceptibility Testing of *Klebsiella pneumoniae* Clinical Isolates Using the Rapid SYBR Green I/PI Staining Method

**Figure 5:**
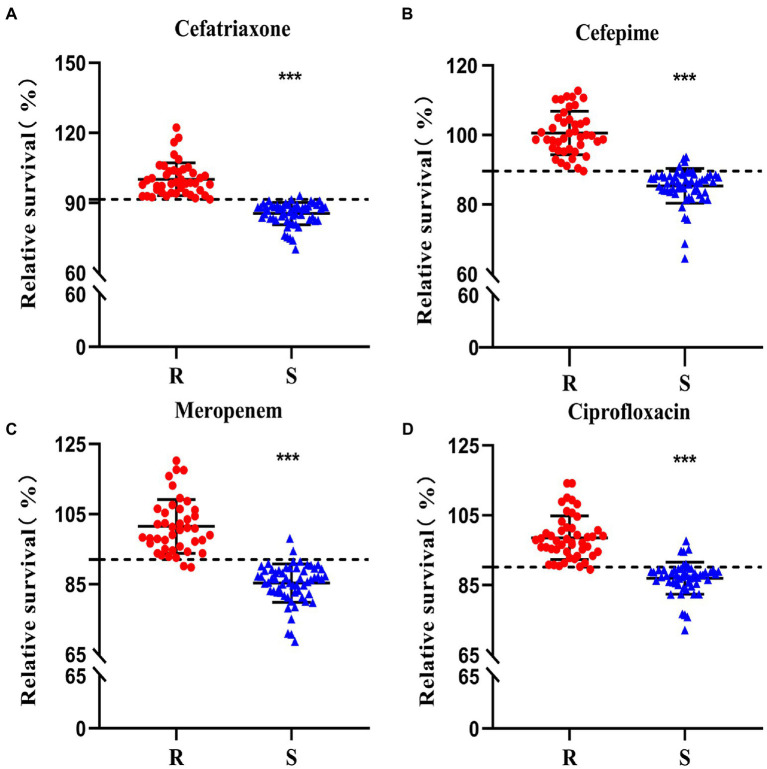
Antibiotic resistance of a total of 100 clinical isolates to ceftriaxone **(A)**, cefepime **(B)**, meropenem **(C)**, and ciprofloxacin **(D)** evaluated using the SYBR Green I/PI staining. The horizontal dashed lines (---) indicate the cutoff values of the resistance determination based on the SYBR Green I/PI staining. Strains identified as resistant (R) and sensitive (S) clinical strains identified in the conventional susceptibility tests are shown in red dots and blue triangles, respectively. The symbol ^“***”^ indicates significant differences set at *p* < 0.0005.

**Figure 6:**
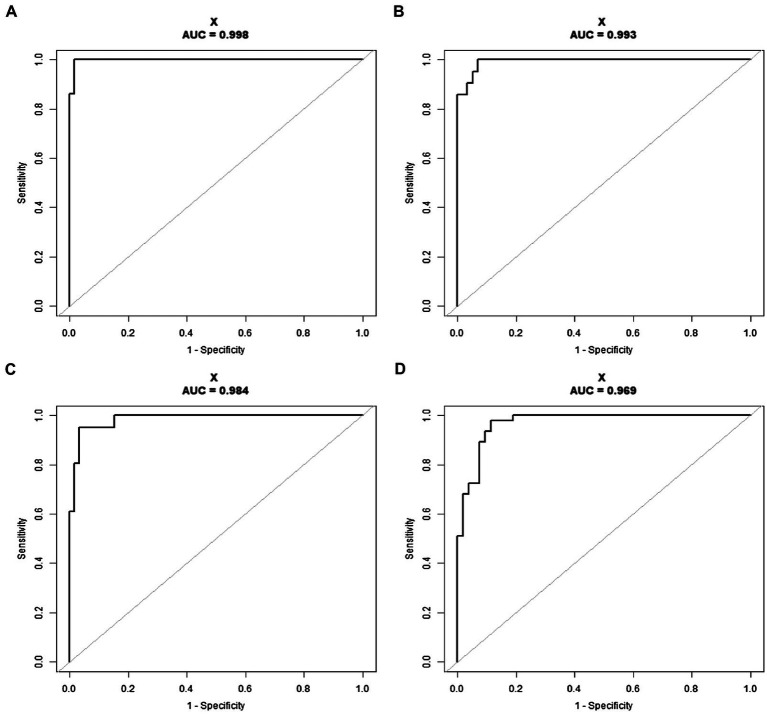
The receiver operating characteristic (ROC) curve based on the antibiotic susceptibility to ceftriaxone **(A)**, cefepime **(B)**, meropenem **(C)**, and ciprofloxacin **(D)** of a total of 100 clinical isolates detected by SYBR Green I/PI staining. AUC is the area under the ROC curve.

To validate the clinical diagnosis and the feasibility of the SYBR Green I/PI staining in antibiotic resistance testing, a total of 100 clinical isolates of *K. pneumoniae* were treated with four types of antibiotics, including 100 μg/ml ceftriaxone, 50 μg/ml cefepime, 50 μg/ml meropenem, and 100 μg/ml ciprofloxacin, respectively. Each strain was tested four times with each of the four antibiotics. Significant differences were observed between the resistant and sensitive clinical strains in response to the treatment of each of the four types of the antibiotics (Figure 5). Based on the cutoff values of the ROC curve (Figure 6), a total of 56/43, 54/42, 57/39, and 47/46 clinical isolates were identified as sensitive/resistant to ceftriaxone, cefepime, meropenem, and ciprofloxacin, respectively.

The diagnostic value of the SYBR Green I/PI AST for resistance detection in clinical strains of *K. pneumoniae* was assessed by the area under the ROC curve (AUC) with the higher value of the AUC indicating better performance of the model. The AUC values of the SYBR Green I/PI AST method for the antibiotics ceftriaxone, cefepime, meropenem, and ciprofloxacin were 0.998, 0.993, 0.984, and 0.969, respectively, indicating extremely high sensitivity and specificity of the prediction model (Figure 6). The results of ROC curves showed that the cutoff values were 91.55, 89.60, 92.07, and 90.15% for ceftriaxone, cefepime, meropenem, and ciprofloxacin, respectively (Figure 6). Therefore, the criteria for distinguishing the antibiotic resistance were set as follows: the RS values more than 91.55, 89.60, 92.07, and 90.15% indicated the intermediate or resistant strains for ceftriaxone, cefepime, meropenem, and ciprofloxacin, respectively. In parallel, the results of the antibiotic resistance testing determined by the rapid SYBR Green I/PI AST method showed 98.25%/100%, 93.10%/100%, 96.61%/95.12%, and 88.68%/97.87% diagnostic efficiencies for sensitivity/specificity for ceftriaxone, cefepime, meropenem, and ciprofloxacin, respectively (Table 1). Overall, the rapid SYBR Green I/PI AST method correctly diagnosed the antibiotic resistance of *K. pneumoniae* clinical isolates with an accuracy of 99% (ceftriaxone), 96% (cefepime), 96% (meropenem), and 93% (ciprofloxacin) compared to those of the conventional susceptibility testing by the Kirby-Bauer method and the broth microdilution method (Table 1).

To address the discrepancy of the rapid SYBR Green I/PI AST method and the conventional susceptibility testing, we retested the antibiotic susceptibility of the 15 strains that gave inconsistent results (Table 1) by Vitek 2 (card 335) and compared the results with those obtained by the SYBR Green I/PI AST method (Table 2). The results showed that in fact one (19111419) of the 15 strains was due to previous error of the conventional AST results such that the results of the testing by the two methods matched, and six of the 15 strains were within 1–2% near the cutoff for resistance, leaving only eight of 15 strains to be inconsistent (Table 2). Thus the revised accuracies of the SYBR Green I/PI AST method were 99% (ceftriaxone), 98% (cefepime), 98% (meropenem), and 96% (ciprofloxacin).

**Table 2:** Retesting of inconsistent strains by the conventional AST and the SYBR Green I/PI staining.

Strain number	Conventional AST*			SYBR Green I/PI (RS%)		
	Cefepime	Ciprofloxacin	Meropenem	Cefepime	Ciprofloxacin	Meropenem
				(cutoff-89.59%)	(cutoff-90.15%)	(cutoff-92.07%)
19084449	≤0.12	≤0.25	≤0.25	S (82.05%)	R (94.76%)	S(70.72%)
19122744	≤0.12	≤0.25	≤0.25	S (82.90%)	R (94.63%)	S (86.72%)
19103537	≤0.12	≤0.25	≤0.25	S (84.07%)	R (97.68%)	S (81.70%)
19071035	≤0.12	≤0.25	≤0.25	S (88.43%)	R (90.69%)	S (90.91%)
19072615	≤0.12	≤0.25	≤0.25	S (83.25%)	R (95.27%)	S (85.53%)
19073775	≤0.12	≤0.25	≤0.25	S (68.75%)	R (91.04%)	S (85.05%)
19102713	≤0.12	≤0.25	≤0.25	R (93.23%)	S (89.75%)	S (79.75%)
19113912	≤0.12	≤0.25	≤0.25	R (90.96%)	S (85.81%)	S (88.805)
19111419	≤0.12	≤0.25	≤0.25	S (87.44%)	S (82.38%)	S (87.34%)
19114139	≤0.12	≤0.25	≤0.25	S (89.53%)	S (86.56%)	R (98.08%)
19114901	≤0.12	≤0.25	≤0.25	S (88.965)	S (84.70%)	R (94.52%)
19122587	≥32R	≥4R	≥16R	R (91.14%)	S (89.48%)	R (113.15%)
19072616	≤0.12	≤0.25	≤0.25	R (92.33%)	S (89.38%)	S (81.28%)
20051129	16R	≥4R	≥16R	R (91.14%)	R (99.08%)	S (89.84%)
20012488	≥32R	≥4R	≥16R	R (110.31%)	R (103.2%)	S (90.15%)

* Vitek 2 N335 was used for retesting of the strains that gave inconsistent results of the conventional microdilution methods and the SYBR Green I/PI staining.

## Discussion

Given the current growing crisis of antibiotic resistance worldwide, it is critical to develop novel, rapid, and reliable diagnostic methods for prompt detection of antibiotic resistance to facilitate the selection of appropriate antibiotic treatment and ultimately to prevent the spread and development of antibiotic-resistant bacteria. In this study, we used the SYBR Green I/PI staining method to successfully test the antibiotic resistance in 100 clinical isolates of *K. pneumoniae* to four types of commonly used antibiotics (i.e., ceftriaxone, cefepime, meropenem, and ciprofloxacin). Results showed that this SYBR Green I/PI staining method could rapidly and accurately determine the antibiotic resistance or sensitivity of these isolates within 60 min in comparison with the conventional AST, which takes 16–24 h.

To expedite the detection of antibiotic resistance in *K. pneumoniae*, we investigated the direct, rapid detection of resistance and sensitivity to ceftriaxone, cefepime, meropenem, and ciprofloxacin in isolates with known antibiotic effects. These four antibiotics are the commonly used drugs for the treatment of *K. pneumoniae* infections, and the mechanisms involved in the bactericidal process include inhibition of bacterial cell wall synthesis and inhibition of bacterial DNA gyrase. In future studies, it is necessary to verify the resistance patterns of these isolates to more types of antibiotics using the SYBR Green I/PI staining method to support the general application of this assay. In order to identify the optimal exposure concentrations of antibiotics, the concentration gradients were chosen according to the MIC values based on the conventional susceptibility testing. Results showed that the optimal concentrations of antibiotics used in a range of tests were significantly higher than the MIC values. This is consistent with the previous study (Feng et al., 2018). These results indicate that for clinical isolates, the application of antibiotics with higher concentrations could shorten the testing time considerably. The choice of the high antibiotic concentrations is determined by pilot experiments with different concentrations of antibiotics using well-defined sensitive and resistant strains. Moreover, in our study, strain resistance was clearly distinguished by extending the antibiotic exposure time from 30 min (Feng et al., 2018) to 40 min. With the extended antibiotic exposure time, our assay based on SYBR Green I/PI staining is still 1–3 h faster than those (multiplex fluidic chip and resazurin with thin-film platinum bio-electrode) reported previously which take about 4 h (Mishra et al., 2019; Wistrand-Yuen et al., 2020).

A similar antibiotic resistance detecting method in *K. pneumoniae* was reported recently based on the PI/Alamar blue double staining (Choi et al., 2020) with low sensitivity and diagnostic specificity of *K. pneumoniae* (i.e., sensitivity rate of 87.5%). Our results revealed high diagnostic sensitivities of four antibiotics (ceftriaxone, cefepime, meropenem, and ciprofloxacin) based on the SYBR Green I/PI staining as 98.25, 93.10, 96.61, and 88.68%, respectively, and concordance rates of 99, 96, 96, and 93% with the conventional growth-based MIC or K-B testing, respectively. Despite the promising results of the SYBR Green I/PI AST method, we noted some discrepancy in AST results for a small number of strains by the SYBR Green I/PI AST method compared with the conventional susceptibility testing methods (Table 1). This is probably partly due to the presence of strains with RS values close to cutoff (Table 2) or low-level resistance or individual strain differences, whereas the misdiagnosis of sensitive strains may be due to the generation of mutations during repeated subculture. Therefore, further optimization, such as adjusting the inoculum size, the concentration of antibiotics, and the cutoff values for resistance, will be needed to perfect the accuracy of the rapid SYBR Green I/PI AST method. The conventional AST is reliable but time-consuming which requires a long time, i.e., 16–24 h, to generate the testing results. Some rapid and high-throughput methods are relatively expensive and often require dedicated personnel (Livermore et al., 2002; Sauget et al., 2014; Trip et al., 2015; Saint-Ruf et al., 2016; Choi et al., 2020), limiting their routine and wide applications in practice. The SYBR Green I/PI staining used in this study provides an easy, fast, and reliable method to determine the antibiotic resistance in clinical isolates of *K. pneumoniae* to four types of commonly used antibiotics. Future studies should include other antibiotics, such as aminoglycosides, so the SYBR Green I/PI AST method becomes more useful. One limitation of this study is that we investigated only the previously identified isolates and not intermediate resistant strains, making it impossible to validate the application of this rapid SYBR Green I/PI AST method to such strains. Further studies including a wide range of clinical isolates of other significant bacterial pathogens are needed to validate the wide application of this method in clinical settings. Application of this rapid SYBR Green I/PI AST method should help to save lives and reduce the empirical use of antibiotics and ultimately the health and economic burdens of antibiotic resistance.

## Conclusion

In summary, we validated the accuracy and feasibility of the rapid SYBR Green I/PI AST method for detecting antibiotic resistance to four types of commonly used antibiotics (ceftriaxone, cefepime, meropenem, and ciprofloxacin) in a total of 100 clinical isolates of *K. pneumoniae* as rapidly as 60 min. In order to further optimize the testing conditions and the evaluation criteria, the future clinical studies with other antibiotics and pathogens are necessary to support the use of the promising ultra-rapid antibiotic resistance detection assay.

## Data Availability Statement

The raw data supporting the conclusions of this article will be made available by the authors, without undue reservation.

## Author Contributions

YW and YiZ designed the experiments and revised the manuscript. YaZ performed the experiments and wrote the manuscript. JW performed the experiments. CS and YJ revised the manuscript. WF and JS critically read the manuscript. All authors contributed to the manuscript and approved the submitted version.

### Conflict of Interest

The authors declare that the research was conducted in the absence of any commercial or financial relationships that could be construed as a potential conflict of interest.
